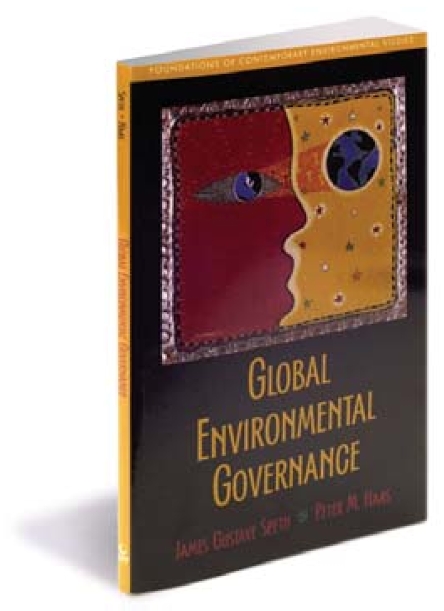# Global Environmental Governance

**Published:** 2007-12

**Authors:** Jacobo Finkelman

**Affiliations:** Jacobo Finkelman is an independent consultant on environment and public health. He has held senior positions with the PanAmerican Health Organization/World Health Organization (PAHO/WHO) as Head of the Epidemiology Surveillance Unit in Washington, DC, and was director of the PanAmerican Center for Human Ecology and Health, PAHO/WHO Country Representative in Guatemala (1993–1998), Brazil (1998–2004), and Mexico (2004–2006)

By James Gustave Speth and Peter M. Haas

Washington, DC:Island Press, 2006. 179 pp. ISBN: 1-59726-081-9, $40

*Global Environmental Governance* provides a very eloquent and concise statement of the key international events and milestones, as well as the most relevant concepts and philosophies that have influenced the global environmental agenda over the past decades.

James Gustave Speth and Peter M. Haas are two well-respected figures, widely recognized for their contributions to the debate of environmental issues. In this book, they suggest a series of innovative proposals on how to improve the world environmental governance to confront one of the most complex problems that humanity is facing.

In the initial chapters, the authors discuss some of the main environmental challenges and their implications for human well-being as well as the associated driving forces involved in each of them. They analyze the overall demographic and political context that industrialized and developing countries are facing, and they put into perspective the potentials and limitations of technological solutions to current and emerging environmental problems. They also address some of the difficult dilemmas directly involved with the enhancement of effective global governance, particularly how the current principles, concepts, and practices of sovereign nations are linked and interrelated with environmental issues in an increasingly interdependent and globalized world. Within this context, they provide a quick overview of the structural complexity of United Nations system and its specialized agencies, and they argue in favor of expanding the competencies of the United Nations Environment Program.

Speth and Haas present very directly some of the inconsistencies and gaps between the proposals emerging from the most relevant environmental international conferences and their practical outcomes, starting with 1972 Stockholm Conference on Human Environment. They share some of the enthusiasm and optimism that characterized the 1992 United Nations Conference on Environment and Development held in Rio de Janeiro, and the disappointment of the lead-up to the World Summit for Sustainable Development held in Johannesburg in 2002.

The authors provide a systematic rather than a reductionist analysis of what has worked and how scientific knowledge has provided the substance for consensus building among nations. This has produced some environmental improvements, such as the reduction and control of chlorofluorocarbons and other chemicals that deplete the ozone layer, through the Montreal Protocol. The authors also present well-structured arguments of other international initiatives and negotiations that have not been as successful, and, as a result, many environmental problems continue to worsen, almost always as a consequence of economic and political interests promoted by special interest groups.

The authors, like many others, recognize the importance of unresolved social problems that have translated into unacceptable inequities. These include realities such as the increasing concentration of wealth, the lack of justice and ethics that afflict many sectors of society, and the slow and limited progress toward participatory democracy in several nations. Greed on one hand and poverty and ignorance on the other are truly the perverse driving forces that hold the future of our civilization in the balance.

Despite all the obstacles and setbacks, the authors are optimistic and give us hope. They suggest that thorny issues such as the conflicting views among some countries on how to reach consensus on reducing greenhouse gas emissions will be bridged, and that new perceptions of the severity and urgency of global environmental change, particularly in the United States and other countries, will cause leaders to adopt positions more in keeping with the growing body of scientific evidence, thereby incorporating major corporations as proactive and socially responsible partners. These new scenarios, among other strategic issues, are essential in the analysis of the possible environmental, political, economic, and social implications associated with the production of so-called biofuels and not only with the reduction of dependency on the importation of oil to industrialized countries.

This book will be a valuable reference for both public policy makers and decision makers, as well as for scholars and academics interested in the environment, sustainability, and the future of humankind. It is without a doubt one of the best books I have read on global environmental governance.

## Figures and Tables

**Figure:**